# Anti-biofilm Properties of the Fecal Probiotic Lactobacilli Against *Vibrio* spp.

**DOI:** 10.3389/fcimb.2018.00120

**Published:** 2018-04-24

**Authors:** Sumanpreet Kaur, Preeti Sharma, Namarta Kalia, Jatinder Singh, Sukhraj Kaur

**Affiliations:** ^1^Department of Microbiology, Guru Nanak Dev University, Amritsar, India; ^2^Department of Molecular Biology and Biochemistry, Guru Nanak Dev University, Amritsar, India

**Keywords:** *Vibrio*, anti-biofilm, biofilm-dispersion, lactobacilli, probiotic, antimicrobial

## Abstract

Diarrheal disease caused by *Vibrio cholerae* is endemic in developing countries including India and is associated with high rate of mortality especially in children. *V. cholerae* is known to form biofilms on the gut epithelium, and the biofilms once formed are resistant to the action of antibiotics. Therefore agents that prevent the biofilm formation and disperse the preformed biofilms are associated with therapeutic benefits. The use of antibiotics for the treatment of cholera is associated with side effects such as gut dysbiosis due to depletion of gut microflora, and the increasing problem of antibiotic resistance. Thus search for safe alternative therapeutic agents is warranted. Herein, we screened the lactobacilli spp. isolated from the fecal samples of healthy children for their abilities to prevent biofilm formation and to disperse the preformed biofilms of *V. cholerae* and *V. parahaemolyticus* by using an *in vitro* assay. The results showed that the culture supernatant (CS) of all the seven isolates of *Lactobacillus* spp. used in the study inhibited the biofilm formation of *V. cholerae* by more than 90%. Neutralization of pH of CS completely abrogated their antimicrobial activities against *V. cholera*, but had negligible effects on their biofilm inhibitory potential. Further, CS of all the lactobacilli isolates caused the dispersion of preformed *V. cholerae* biofilms in the range 62–85%; however, pH neutralization of CS reduced the biofilm dispersal potential of the 4 out of 7 isolates by 19–57%. Furthermore, the studies showed that CS of none of the lactobacilii isolates had antimicrobial activity against *V. parahaemolyticus*, but 5 out of 7 isolates inhibited the formation of its biofilm in the range 62–82%. However, none of the CS dispersed the preformed biofilms of *V. parahaemolyticus*. The ability of CS to inhibit the adherence of *Vibrio* spp. to the epithelial cell line was also determined. Thus, we conclude that the biofilm dispersive action of CS of lactobacilli is strain-specific and pH-dependent. As *Vibrio* is known to form biofilms in the intestinal niche having physiological pH in the range 6–7, the probiotic strains that have dispersive action at high pH may have better therapeutic potential.

## Introduction

Cholera is an acute diarrheal infection caused by the ingestion of food or water contaminated with the cholera toxin-producing Gram-negative pathogen *Vibrio cholerae*. It has been the cause of 7 pandemics since 1,871, and still remains a major public health issue in more than one-third of the countries due to poor sanitation facilities and lack of safe drinking water (Morris and Acheson, 2003). The annual occurrence of cholera in 69 cholera-endemic countries is estimated to be 2.9 million that results in 95,000 deaths each year (Ali et al., 2015). It is an endemic disease in India and can be fatal if remained unmanaged. *V. parahaemolyticus* that secrets haemolysin is also known to cause gastroenteritis if consumed at high doses. The extensive use of antibiotics for the treatment of diarrhea has led to the emergence of drug resistance in *V. cholerae* (Kitaoka et al., 2011). Also the use of antibiotics can disrupt the homeostasis of the gut by killing the normal gut flora. Therefore, there is a need for safe alternative therapeutics to combat gut-bacterial infections. One of the safe alternative therapeutic agents is probiotics. Probiotics are live microorganisms which confer health benefits on the host when administered in adequate amounts (FAO/WHO, 2001). *Lactobacillus* spp. is the most widely accepted probiotic because of their GRAS (generally regarded as safe) status. Lactobacilli are rod-shaped Gram-positive, facultative anaerobes, comprising roughly 0.01% of the microbiome in the gastrointestinal tract (GIT) of humans (Harmsen et al., 2002) and range between 10^7^-10^8^ cells/gm of feces (Rinttilä et al., 2004). In GIT, they are known to possess health-promoting effects such as maintaining normal intestinal homeostasis by inhibiting the colonization of pathogens and modulating the immune responses (Lebeer et al., 2008; Kemgang et al., 2014). The use of probiotics for the treatment of gut-associated health disorders has yielded positive results as demonstrated by various clinical trials (Culligan et al., 2009). Meta-analysis of clinical trials showed the efficacy of lactobacilli probiotics for the treatment of antibiotic-induced diarrhea (D'Souza et al., 2002; McFarland, 2006), antibiotic-induced *Clostridium difficile* infection (McFarland, 2006), and reduction in the duration of rotavirus diarrhea (Huang et al., 2002; Ahmadi et al., 2015). However, the human clinical trial of probiotics against cholera was not successful (Mitra and Rabbani, 1990). *V. cholerae* is a biofilm-forming pathogen. It is known to form strong biofilms on the epithelial lining of gut in both mice (Millet et al., 2014) and humans (Yamamoto and Yokota, 1988). The biofilms play an important role in the pathogenesis of cholera (Fong et al., 2010; Almagro-Moreno et al., 2015). Also once the biofilms are formed, they resist the action of both immune defenses and antibiotics, and are also responsible for the recurrent nature of the infection. Therefore the probiotic strain having both antimicrobial and anti-biofilm properties may be expected to be therapeutically more effective. Some of the anti-biofilm agents effective against *Vibrio* spp. have been reported in the literature (Sambanthamoorthy et al., 2011; Sayem et al., 2011; Warner et al., 2015), but they do not have any antimicrobial properties against planktonic cells and thus are administered along with the conventional antibiotics. The antimicrobial probiotics having biofilm-dispersive properties can yield better clinical benefits for the treatment of diarrhea due to *Vibrio* spp as they may be used as stand-alone therapeutic agents. The *in vitro* antimicrobial activity of lactobacilli strains against different *Vibrio* spp. (Koga et al., 1998), *V. parahaemolyticus* (Shokryazdan et al., 2014; Chimchang et al., 2015) and *V. cholerae* (Petrova and Petrov, 2011) has been demonstrated by various workers. However the ability of lactobacilli in inhibiting the biofilm of *Vibrio* spp. has not been reported. With this in the background, we isolated lactobacilli from the fecal samples of healthy children and studied the antimicrobial and anti-biofilm activities of cell free culture supernatant (CS) of lactobacilli. The probiotic properties of the selected isolates were also studied with an idea to develop them as indigeneous probiotic strain.

## Materials and methods

### Microorganisms and growth conditions

For the isolation of lactobacilli, fecal samples were collected from 32 healthy children of age group ranging from 2 to 13 yrs after taking the written informed consent of their parents. The study was approved by the Institutional Human Ethics Committee. The stool sample weighing approximately 1 g was collected in thioglycollate broth (HiMedia laboratories, Mumbai, India) and incubated for 4 h at 37°C in anaerobic jars having 5% carbon dioxide (CO_2_). Thereafter, 10-fold serial dilutions of the broth were plated onto De Man Rogosa and Sharpe (MRS; HiMedia) agar plates and incubated at 37°C under anaerobic conditions in anaerobic gas jars. Bacterial colonies with different morphologies were selected and preserved in 20% (v/v) glycerol (HiMedia)-containing MRS broth at −80°C. The lactobacilli were identified by Gram-positive staining and catalase-negative phenotype. For experimental purposes, the lactobacilli were cultured in MRS medium from the frozen stocks and propagated twice before use.

The various pathogenic indicator strains used in this study were *V. cholerae* strain 0139 MTCC 3906*, Salmonella enterica* Typhimurium MTCC 733*, Listeria monocytogenes* MTCC 657*, Escherichia coli* MTCC 119, *Shigella flexeri* MTCC 1457, *V. parahaemolyticus* MTCC 451, and *Staphylococcus aureus* MTCC 96. These strains were procured from Microbial Type Culture Collection, Institute of Microbial Technology, Chandigarh, India. The fungal indicator strain *Candida* spp. was procured from the Department of Microbiology, Government Medical College, Amritsar. All the pathogenic bacteria were cultured in brain heart infusion (BHI) broth (HiMedia) and *Candida* spp. was cultured in Sabouroud dextrose broth (HiMedia) at 37°C under aerobic conditions. All the cultures were stored at −80°C in broth supplemented with 20% glycerol.

### Characterization of the lactobacilli isolates

The lactobacilli isolates were characterized by using 16S rDNA sequencing and biochemical tests. For 16S rDNA sequencing, the genomic DNA of lactobacilli was isolated according to the method described by Moore et al. (2004). Following DNA isolation, 16S rDNA was amplified by PCR using universal primers-27F Forward: 5′-AGAGTTGATCCTGGCTCAG-3′ and 1492P Reverse: 5′-TACGGCTACCTTGTTACGACTT-3′. DNA amplification was carried out in 0.2 ml PCR tubes by using master cycle personal (Eppendorf Hamburg, Germany). The PCR reaction mixture (50 μl) consisted of 25 μl of 2X PCR master mix (3B Black Bio Biotech India, Ltd.,), 1 μl of each primer (Bioserve Biotechnologies Pvt. Ltd., India), 5 μl of template DNA, and 18 μl of nuclease free water. Initial denaturation of DNA was done at 95°C for 4 min, followed by 32 cycles of amplification comprising a denaturation step for 1 min at 95°C, annealing at 56°C for 1 min 30 s and extension at 72°C for 1 min. Reactions were completed with 10 min elongation at 72°C followed by cooling to 4°C. The PCR products were analyzed by electrophoresis on a 1.5% agarose gel at 100 V for 45 min against 100 bp step ladder. The bands were visualized with bio imaging system (Gene Genius gel imaging System, Syngene Bioimaging Private Ltd., India). The partial sequences of 16S rDNA were obtained and the isolate identified by aligning in the software BLAST version 2. The 16s rDNA sequences obtained have been submitted to NCBI and their accession no. were obtained (Table 1).

**Table 1:** The GenBank accession numbers of fecal lactobacilli isolates characterized by using 16S rDNA sequencing.

**Isolate**	**Genus species**	**Accession no**.
L13	*Lactobacillus spp*.	KY780504
L14	*Lactobacillus plantarum*	KY582835
L18	*Lactobacillus spp*.	KY770976
L32	*Lactobacillus fermentum*	KY770983
S30	*Lactobacillus spp*.	KY780503
S45	*Lactobacillus pentosus*	KY780505
S49	*Lactobacillus spp*.	KY770966

### Determination of the antimicrobial activity of CS of lactobacilli isolates

The agar well diffusion assay was used to determine the antimicrobial activities of CS of lactobacilli isolates grown in MRS broth (Reinheimer et al., 1990; Gonzalez et al., 2007). To prepare the CS, lactobacilli were cultured in MRS broth for 16 h at 37°C and the broth was centrifuged at 9,000 g for 10 min at 4°C. The supernatant was then filter sterilized using syringe filters having pore size of 0.2 μm and stored at 4°C till further use.

For performing agar well diffusion assay, the indicator strains were grown at 37°C in the appropriate medium till an optical density (OD_595_) of 0.2 is obtained. Following incubation, 100 μl of the indicator pathogen or commensal culture was spread onto suitable agar plates and 6 mm wells were cut into the agar plates by using sterile well-borer. An aliquot (100 μl) of CS was poured into wells and plates were placed at 4°C for 4 h to allow diffusion of CS into agar. Then the plates were incubated at 37°C for 24 h and the diameter of zone of inhibition around each well was measured in millimeters.

To nullify the effect of reduced pH on antimicrobial activity, the pH of CS was adjusted to 6.5 by using 1 N NaOH (HiMedia) and antimicrobial activity was similarly determined.

### Growth curve

The growth curves of *V. cholerae* and *V. parahaemolyticus* were made in BHI medium supplemented with lyophilized MRS (as control) or pH-neutralized/non neutralized CS of lactobacilli isolates at the concentrations of 45 mg/ml. The overnight *Vibrio* cultures were grown in BHI and diluted to 0.1 OD_595_ before inoculating in BHI supplemented with MRS or CS at the concentration 45 mg/ml and incubated at 37°C for 48 h in the total volume of 220 μl in 96-well polystyrene plates in triplicates. Absorbance at the wavelength 595 was measured after every 4 h on the microplate reader (Biorad) till 48 h.

### The effect of CS on the biofilm formation by *Vibrio* spp.

The effect of pH non-neutralized CS of lactobacilli isolates was determined on the biofilm formation by both *V. cholerae* and *V. parahaemolyticus*, whereas, the effects of pH neutralized CS (pH set to 6.5 by using 1 N NaOH) was tested only on the biofilm formation by *V. cholerae*. The biofilm formation was determined by using modified crystal violet assay (Sharma et al., 2015) in a 96-well polystyrene microtiter plate (Tarsons Product Pvt. Ltd., Kolkata). To initiate biofilm formation, 100 μl of sterile BHI broth was added to each well along with 100 μl of CS or MRS broth (at concentration 45 mg/ml) and 20 μl of overnight grown *Vibrio* spp. having OD_595_ of 0.1. The microtiter plate was incubated at 37°C for 48 h to allow biofilm formation. Following incubation, the non adherent cells were removed by washing the wells gently 3 times with sterile distilled water. The adherent cells were fixed by using 200 μl of methanol (HiMedia) for 15 min and then the plate was emptied and air dried. The fixed biofilms were stained by using 200 μl of 2% crystal violet (HiMedia) in distilled water for 5 min. Excess stain was removed by washing under running tap water till color fades away. The stain was extracted from the adherent cells by using 160 μl of 33% glacial acetic acid (HiMedia) in distilled water and OD_595_ was measured using microplate reader. The experiment was conducted in triplicates. The percentage inhibition was calculated as,

Percentage inhibition = 100–[(OD_595_ of wells in the presence of CS X 100)/ OD_595_ of wells in the presence of MRS].

### Effect of CS of lactobacilli on the dispersal of biofilms of *Vibrio* spp.

The effect of pH non-neutralized CS was determined on the dispersion of preformed biofilm of both *V. cholerae* and *V. parahaemolyticus*; whereas the effect of pH-neutralized CS (pH set to 6.5 by using 1 N NaOH) was studied only on the biofilm of *V. cholera* (Wu et al., 2013). Biofilm of *Vibrio* spp. was developed in 96-well microtiter plate by adding 100 μl of autoclaved BHI broth along with 20 μl of overnight grown *Vibrio* culture having OD_595_ of 0.1. After 24 h incubation at 37°C, non adherent cells were removed by gentle pipetting without disrupting biofilm. CS of lactobacilli (at concentration 45 mg/ml) were added to each well along with 100 μl BHI broth. In the control wells instead of CS 100 μl of autoclaved MRS broth was added. The plates were incubated at 37°C for 48 h. The experiment was conducted in triplicates. After specified incubation, quantification of biofilm formed was done as described previously.

### Bacterial adhesion assays with HCT-15 cell line

The binding of *Vibrio* spp. to the intestinal cell line HCT-15 in the presence and absence of CS of lactobacilli isolates was determined. In separate set of experiments the binding of lactobacilli to HCT-15 was also determined. The intestinal cell line HCT-15 was cultured in Roswell Park Memorial Institute (RPMI)-1640 (Sigma-Aldrich) medium supplemented with 10% fetal calf serum (Biological Industries, USA) on the autoclaved glass coverslips kept in 60 mm petridishes and incubated at 37°C in 5% CO_2_-containing atmosphere. After the cells formed 50% confluent monolayer, they were washed twice with PBS (pH 7.2) and the viable overnight grown cells of *Vibrio* spp. or lactobacilli suspended in 4 ml of RPMI were added to the petridishes at the multiplicity of infection 1:100. For determining the effect of CS, the cells of vibrio suspended in 2 ml of RPMI along with 2 ml of CS of lactobacilli were added to the cell line-containing petridishes. After incubation for 1 h at 37°C, the dishes were washed four times with PBS (pH 7.2) to remove the unbound bacteria. The cells were fixed with 3 ml of methanol for 5–10 min at room temperature. The cells were air dried and stained with 3 ml of Giemsa stain solution (HiMedia) by incubating at room temperature for 30 min. The dishes were washed until no color was observed in the washing solution, dried in an incubator at 37°C overnight, and examined microscopically under oil immersion. The adherent lactobacilli in 25 random microscopic fields were counted for each test. Bacterial strains were scored as non-adhesive when fewer than 40 bacteria were present in 25 fields, adhesive with 41 to 100 bacteria in 25 fields, and strongly adhesive with more than 100 bacteria in 25 fields. The adhesion assay was performed in duplicate.

Simultaneously the other set of HCT-15-containing petridishes were subjected to the treatment with lysis solution (0.05% trypsin-EDTA) for 30 min at 37°C in order to lyse and detach the cells, and thereafter plated onto BHI agar plates for CFU counting.

### Estimation of lactic acid production

The percentage of lactic acid produced by *Lactobacillus* isolates in CS was determined by titration method. Briefly, the overnight grown lactobacilli culture in MRS broth was centrifuged at 9,000 g for 10 min at 4°C in cooling centrifuge. One ml of 0.5% phenolphthalein indicator dissolved in 50% ethanol was added to 9 ml CS and then the solution was titrated using 1N NaOH (SRL) till the appearance of light pink color. The percentage lactic acid in the CS was equal to the percent NaOH used to neutralize the acidity.

### Probiotic potential of lactobacilli

#### Gastric juice tolerance and bile tolerance

The overnight grown lactobacilli were pelleted down by centrifugation at 9,000 g at 4°C for 10 min. The pellet was resuspended in simulated gastric juice having 2 g/l NaCl (HiMedia), 3.2 g/l pepsin and pH adjusted to 2.5 with conc. HCl (HiMedia). The suspension was incubated at 37°C for 3 h. Cells suspended in 1X PBS buffer (pH-7.2) was used as control. The viabilities of bacterial cells were evaluated by spreading onto MRS agar plates.

To determine bile tolerance, the serially diluted cultures were spread onto MRS agar plates supplemented with 0.3% oxgall (HiMedia). MRS agar plates without oxgall were used as control. The plates were incubated at 37°C in anaerobic jars. The colonies were counted after 24 h.

#### Biofilm formation by lactobacilli

Biofilm-forming abilities of lactobacilli isolates was determined in 96-well microtiter plate in MRS broth set at two different pH−4 and 6 by using crystal violet assay. Briefly, 135 μl of the autoclaved MRS broth was added to each well along with 15 μl of overnight grown lactobacilli culture having OD_595_ of 0.1. The microtiter plates were incubated at 37°C for different time periods−24, 48, and 72 h and the non adherent bacteria were removed by gently washing 3 times with the autoclaved distilled water. The biofilms were then fixed and stained as described previously. The sterile MRS broth was used as control. The experiment was performed in triplicates.

Based on obtained OD, strains were classified as –

1. Non-biofilm producers OD ≤ OD_c_.
2. Weak biofilm producers OD_c_ < OD ≤ 2OD _c_.
3. Moderate biofilm producers 2OD_c_ < OD ≤ 4OD _c_.
4. Strong biofilm producers 4OD_c_ < OD.
      OD: OD of experimental well having lactobacilli cells in MRS broth.
      OD_c_: OD of control well having only MRS broth.

#### Autoaggregation assay

Overnight grown bacterial cells were centrifuged at 9,000 g for 10 min at 4°C. The CS was discarded and pellet was diluted in PBS buffer (pH-7.2) to give final OD_595_ of 1. The suspension of lactobacilli was incubated at 37°C for 4 and 8 h. After incubation period, 1 ml of the suspension from the top of the tube was removed and its absorbance was determined at 595 nm. Autoaggregation percentage was determined using equation: (1–A_t_/A_0_) × 100; where A_t_ is absorbance of suspension at different time points and A_0_ is absorbance at beginning of experiment (0 h). The experiment was performed in triplicates.

### Antibiotic susceptibility test

The antibiotic susceptibility profiles of all the lactobacilli isolates was analyzed by using Kirby-Bauer diffusion test (Bauer et al., 1966). The antibiotic discs (HiMedia): tetracycline, streptomycin, ciprofloxacin, moxifloxacin, gentamycin, ampicillin, pencillin, vancomycin, clindamycin, kanamycin, and erythromycin were used in this study. The classification as “susceptible,” “intermediate,” or “resistant” was based on the European Food Safety Authority (EFSA)-recommended breakpoints for diameters of zone of inhibition (EFSA, 2012).

### Effect of commercially available drugs on the growth of lactobacilli

The agar well diffusion assay was used to study the effect of commercially available drugs on growth of lactobacilli isolates. The various tablet formulations used in this study were–paracetamol(500 mg/ml), diclofenac (10 mg/ml), nimugesic (20 mg/ml), ibuprofen (120 mg/ml), cetrizine HCl (2 mg/ml), and lansoprazole(4 mg/ml).

The lactobacilli were grown at 37°C for 18 h in MRS broth. Following the incubation period, 100 μl of culture was spread onto MRS agar plates (HiMedia). Using sterile well-borer, wells were cut onto agar plates. An aliquot (100 μl) of various drugs were poured in wells and plates were placed at 4°C for diffusion. After 4 h, plates were incubated at 37°C overnight. The diameter of zone of clearance was noted in millimeters.

### Statistical analysis

The results from at least three independent experiments represented as mean ± SD (standard deviation). Comparisons were performed by using unpaired Student's *t*-test in GraphPad Prism 5.04 software. In autoaggregation experiments, comparisons were performed by using paired Students's *t*-test.

## Results

### Characterization of the lactobacilli isolates by 16S rDNA sequencing

On the basis of antimicrobial activity, 7 lactobacilli isolates were selected and characterized by 16S rDNA sequencing. BLAST analysis showed that L14 was 99% similar to *L. plantarum*, L32 and S45 was 98% similar to *L. fermentum* and *L. pentosus* respectively. L13, L18, S30, and S49 had <97% sequence matching with any known species and thus appear to be novel strains (Table 1).

### Determination of antimicrobial activity of lactobacilli isolates against test organisms

The well diffusion assay was used to determine the antimicrobial activity of *Lactobacillus* isolates. Out of 55 isolates, 7 isolates had antimicrobial activities against *V. cholerae, E. coli* and *S. enterica*. Five of the isolates had antimicrobial activity against *L. monocytogenes*, 4 of them against *Sh. flexeri*, and only 3 isolates (S30, S45, and S49) inhibited *St. aureus* (Table 2). None of the CS had any antimicrobial activities against the pathogens *V. parahaemolyticus* and *C. albicans*. Also, the CS of all the isolates had no antimicrobial activity against the gut commensal lactobacilli isolates when they were tested against each other (Table 2).

**Table 2:** The antagonistic activities of the cell-free culture supernatants of fecal lactobacilli against various pathogenic and commensal indicator strains.

**Indicator strains**	**Zones of inhibition (mm)** ± **S.D**.						
	**L13**	**L14**	**L18**	**L32**	**S30**	**S45**	**S49**
*V. cholerae*	20 ± 0.3	16 ± 0.1	24 ± 0.3	18 ± 0.2	26 ± 0.2	19 ± 0.2	18 ± 0.2
*S. enterica*	11 ± 0.1	11 ± 0.2	6 ± 0.2	12 ± 0.1	9 ± 0.1	8 ± 0.2	9 ± 0.1
*E. coli*	11 ± 0.2	12 ± 0.2	9 ± 0.1	14 ± 0.3	12 ± 0.2	6 ± 0.1	9 ± 0.2
*St. aureus*	–	–	–	–	20 ± 0.2	22 ± 0.2	19 ± 0.1
*L. monocytogenes*	24 ± 0.3	–	25 ± 0.2	–	18 ± 0.1	23 ± 0.3	17 ± 0.1
*Sh. flexeri*	10 ± 0.1	14 ± 0.2	0.8 ± 0.1	–	10 ± 0.2	–	–
*V. parahaemolyticus*	–	–	–	–	–	–	–
*Candida spp*.	–	–	–	–	–	–	–
*Lactobacillus* L13	ND	–	–	–	–	–	–
*Lactobacillus* L14	–	ND	–	–	–	–	–
*Lactobacillus* L18	–	–	ND	–	–	–	–
*Lactobacillus* L32	–	–	–	ND	–	–	–
*Lactobacillus* S30	–	–	–	–	ND	–	–
*Lactobacillus* S45	–	–	–	–	–	ND	–
*Lactobacillus* S49	–	–	–	–	–	–	ND

*ND: Not Done, –: No zone of inhibition, The CS of lactobacilli isolates without pH neutralization were tested for the antimicrobial activities by using agar-gel diffusion assay. The experiment was performed three times in triplicate. The results are expressed as the means ± standard deviations*.

The CS of all the 7 isolates after 16 h of growth in MRS broth had final pH in the range 3–4 (data not shown). Therefore in order to determine whether the antimicrobial property of CS is due to low pH, the pH of CS was set to 6.5 and its antimicrobial activity tested. The antimicrobial activities of all the CS against all the pathogens was completely abrogated after neutralizing the pH to 6.5 (data not shown). Further, *Lactobacillus* spp. is known to produce hydrogen peroxide that exhibits antimicrobial activity against various Gram-negative bacteria (Pridmore et al., 2008). Therefore, to negate the role of hydrogen-peroxide, we treated the CS of all the lactobacilli isolates with catalase before evaluating their antimicrobial activity. Our results showed that the catalase treatment had no effect on the antimicrobial activity of CS (data not shown).

### Effect of CS on the growth kinetics of *V. cholerae* and *V. parahaemolyticus*

**Figure 1:**
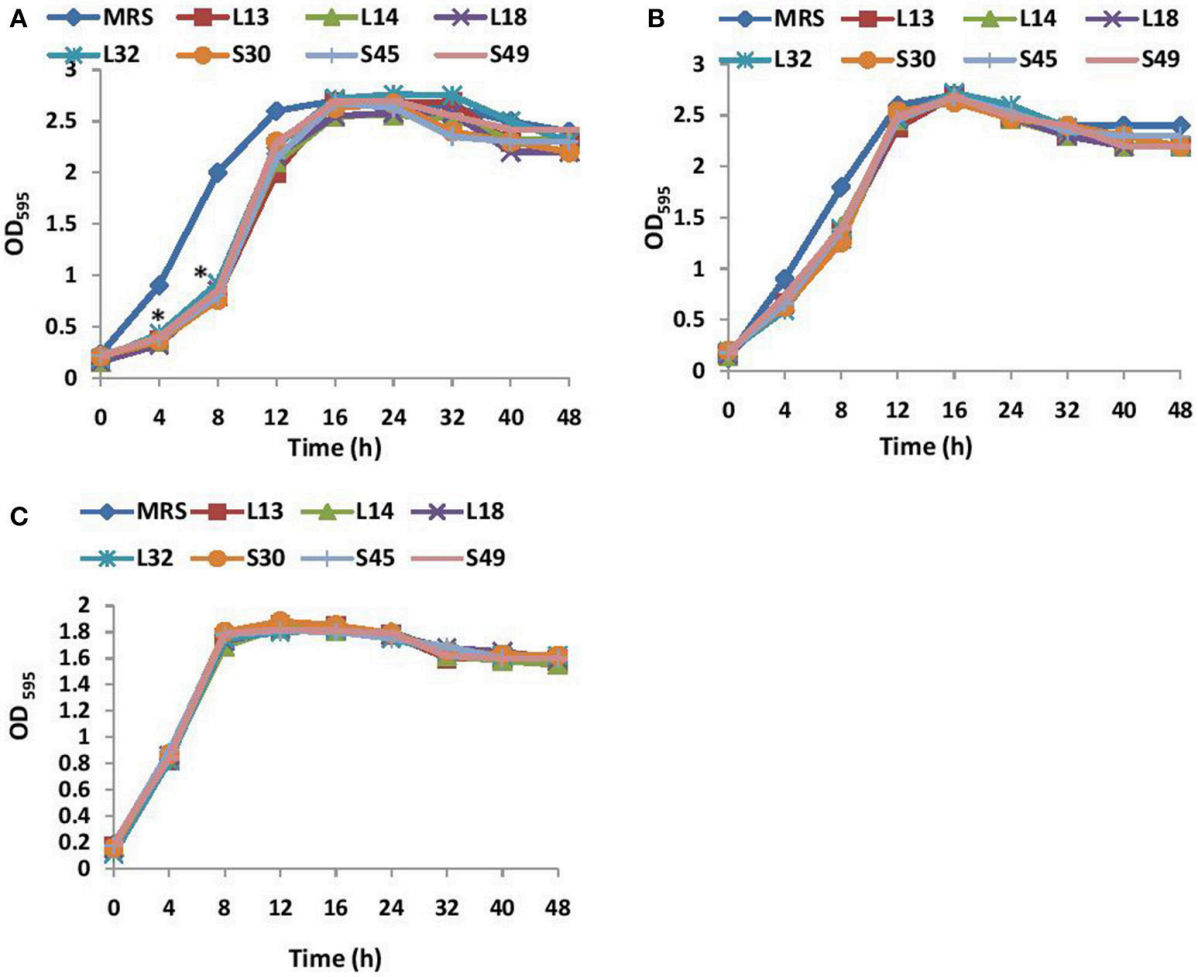
**(A)** Growth curves of *V. cholera* in BHI supplemented with non-neutralized CS and **(B)** pH neutralized CS of lactobacilli strains. **(C)** Growth curve of *V. parahaemolyticus* in BHI supplemented with non-neutralized CS of seven different Lactobacilli strains. ^*^Significance (*p* < 0.001) was measured by using an unpaired Student's *t*-test relative to the growth in BHI supplemented with MRS at 45 mg/ml.

The growth kinetics of *V. cholerae* in BHI media supplemented with non-neutralized and pH neutralized CS was studied. The results showed that the supplementation of BHI growth medium with non-neutralized CS of all the 7 lactobacilli strains significantly (*p* < 0.001) inhibited the growth of *V. cholerae* till 12 h relative to that observed in BHI supplemented with MRS (Figure 1A). Beyond 12 h, no significant differences in the growth kinetics of the wells with and without CS was observed. On the other hand, the supplementation of BHI with pH-neutralized CS of all the seven lactobacilli strains had no significant (*p* < 0.001) effects on the growth kinetics of *V. cholerae* (Figure 1B) at all time points. Similarly the effect of non-neutralized CS of all the lactobacilli strains on the growth of *V. parahaemolyticus* was tested and the results showed that none of the CS inhibited its growth (Figure 1C).

### Effect of CS of lactobacilli on biofilm formation of *Vibrio Cholera*

**Figure 2:**
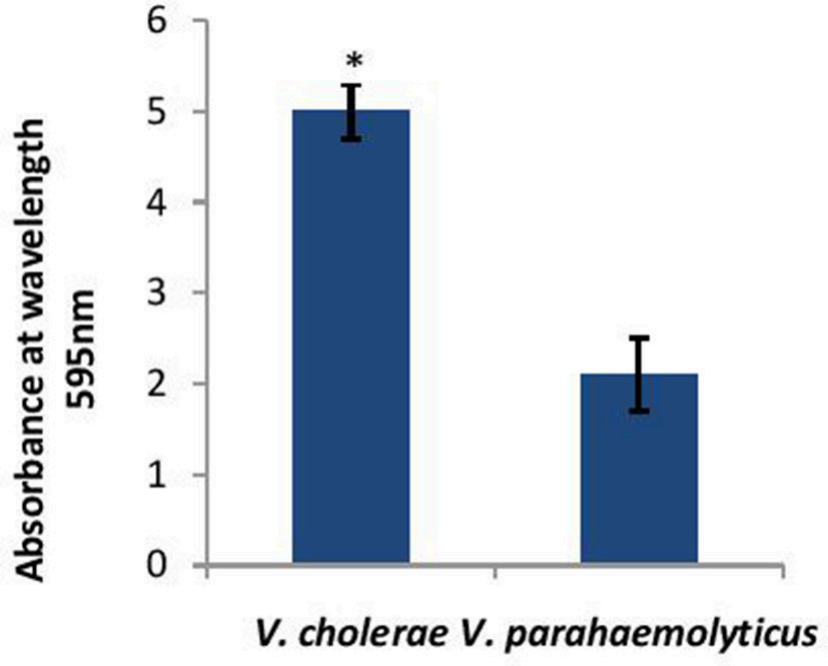
Biofilm forming abilities of *V. cholerae* and *V. parahaemolyticus* evaluated by crystal violet assay performed in microtiter plate. Bars represent the mean and error bars represent standard deviation of three independent experiments. ^*^Significance (*p* < 0.001) was calculated by using an unpaired Student's *t*-test.

Before determining the effect of CS of *Lactobacillus* spp. on the biofilm-forming abilities of both *V. cholerae* and *V. parahaemolyticus*. the biofilm-forming potential of *V. cholerae* and *V. parahaemolyticus* in microtiter plates was determined. As shown in Figure 2, *V. cholerae* formed 2.5 times stronger (*p* < 0.001) biofilms as compared to *V. parahaemolyticus* after 24 h of growth.

**Figure 3:**
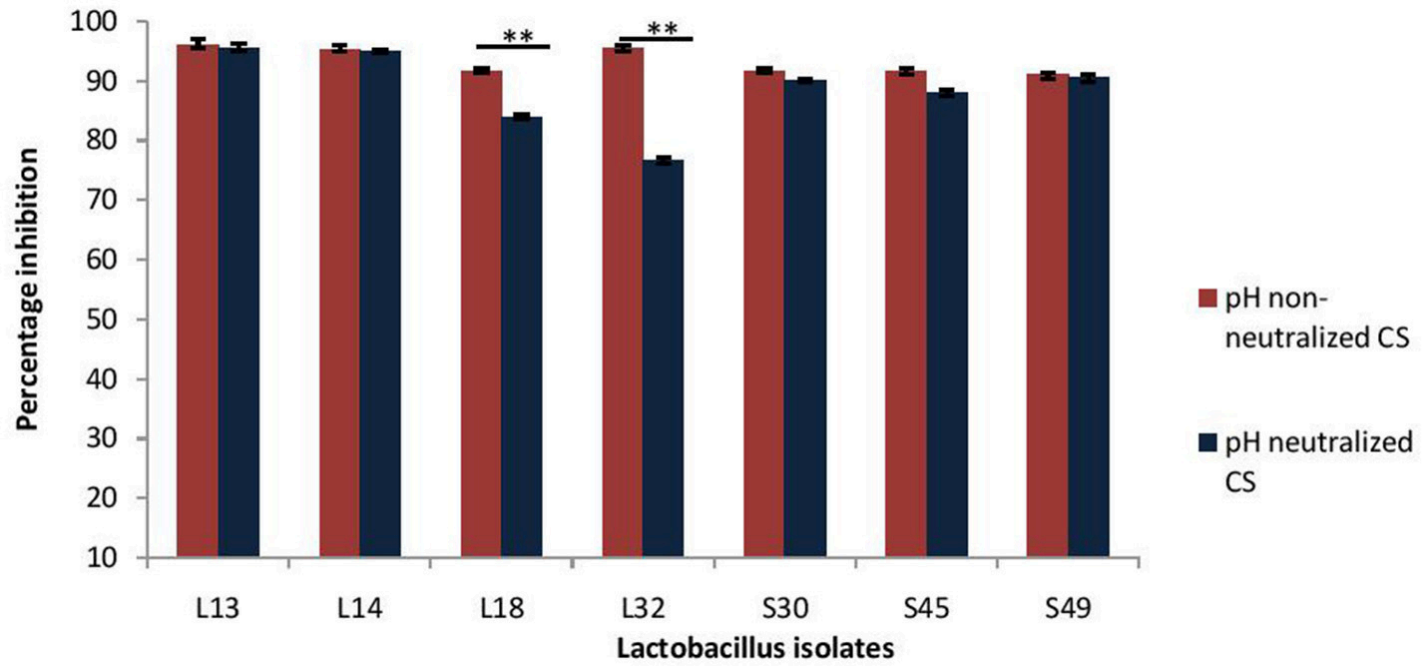
Percentage inhibition of biofilm formation of *V. cholerae* by pH neutralized and non-neutralized CS of fecal *Lactobacillus* isolates evaluated by modified crystal violet assay performed in a microtiter plate. Bars are representative of the mean and error bars are representative of standard deviation of three independent experiments. ^**^Significance (*p* < 0.0001) was measured by using an unpaired Student's *t*-test.

Next, the effect of CS of lactobacilli isolates on the formation of biofilm by *V. cholerae* was evaluated in an *in vitro* assay. The pH non-neutralized CS of all the seven isolates resulted in more than 90% inhibition (Figure 3) of the biofilm formation by *V. cholerae*. Maximum inhibition was observed in case of isolates L13 (96%), L14 (95%), and L32 (95.6%).

Further, as the pH neutralization of CS abrogated its antimicrobial activity, we evaluated the effect of pH neutralized-CS on the biofilm formation by *V. cholerae*. The results showed that the pH neutralized-CS of all the isolates except L32 and L18 resulted in similar inhibition of the biofilm formation by *V. cholerae*. (Figure 3). In case of L32 and L18, the pH neutralization of CS significantly (*p* < 0.0001) reduced their potential to inhibit biofilm formation by 22 and 9%, respectively.

### Effect of CS of lactobacilli on the dispersion of biofilm of *V. cholerae*

**Figure 4:**
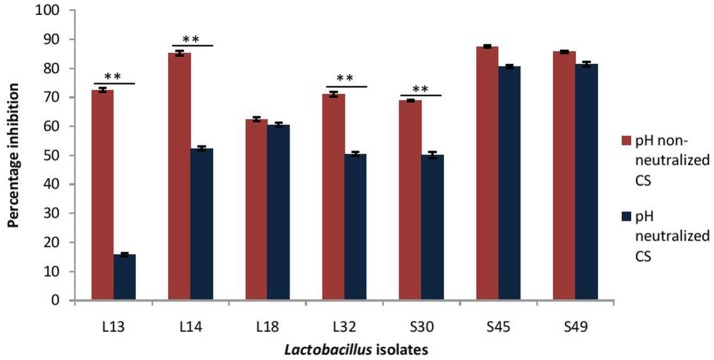
Percentage dispersion of pre-formed biofilm of *V. cholerae* by pH neutralized and non-neutralized CS of fecal *Lactobacillus* isolates evaluated by modified crystal violet assay performed in a microtiter plate. Bars are representative of the mean and error bars are representative of the standard deviation of three independent experiments. ^**^Significance (*p* < 0.0001) was measured by using Student's *t*-test.

As probiotic treatment are prescribed after the infection has established, thus dispersive action of probiotics on *V. cholera* biofilms may impart therapeutic benefits. Herein, the dispersion effect of CS at low pH (3.5) and after pH-neutralization was evaluated on the 24 h old preformed biofilms of *V. cholerae*. The results showed that CS of L14, S45, and S49 resulted in maximum dispersion of 85%, whereas the CS of the other lactobacilli isolates caused dispersion of *V. cholera* biofilms in the range 62–72% (Figure 4).

On adjusting the pH of CS to 6.5 the dispersive effect of the CS of the isolates S45, S49, and L18 was not affected; however, it was significantly (*p* < 0.0001) reduced in the case of four of the isolates viz. L13 (57% reduction), L14 (34%), L32 (19%), and S30 (19%). Thus, the dispersion effect of the CS is affected by pH neutralization in more than 50% of the isolates (Figure 4).

### Effect of CS of lactobacilli on the formation and dispersal of biofilm by *V. parahaemolyticus*

**Figure 5:**
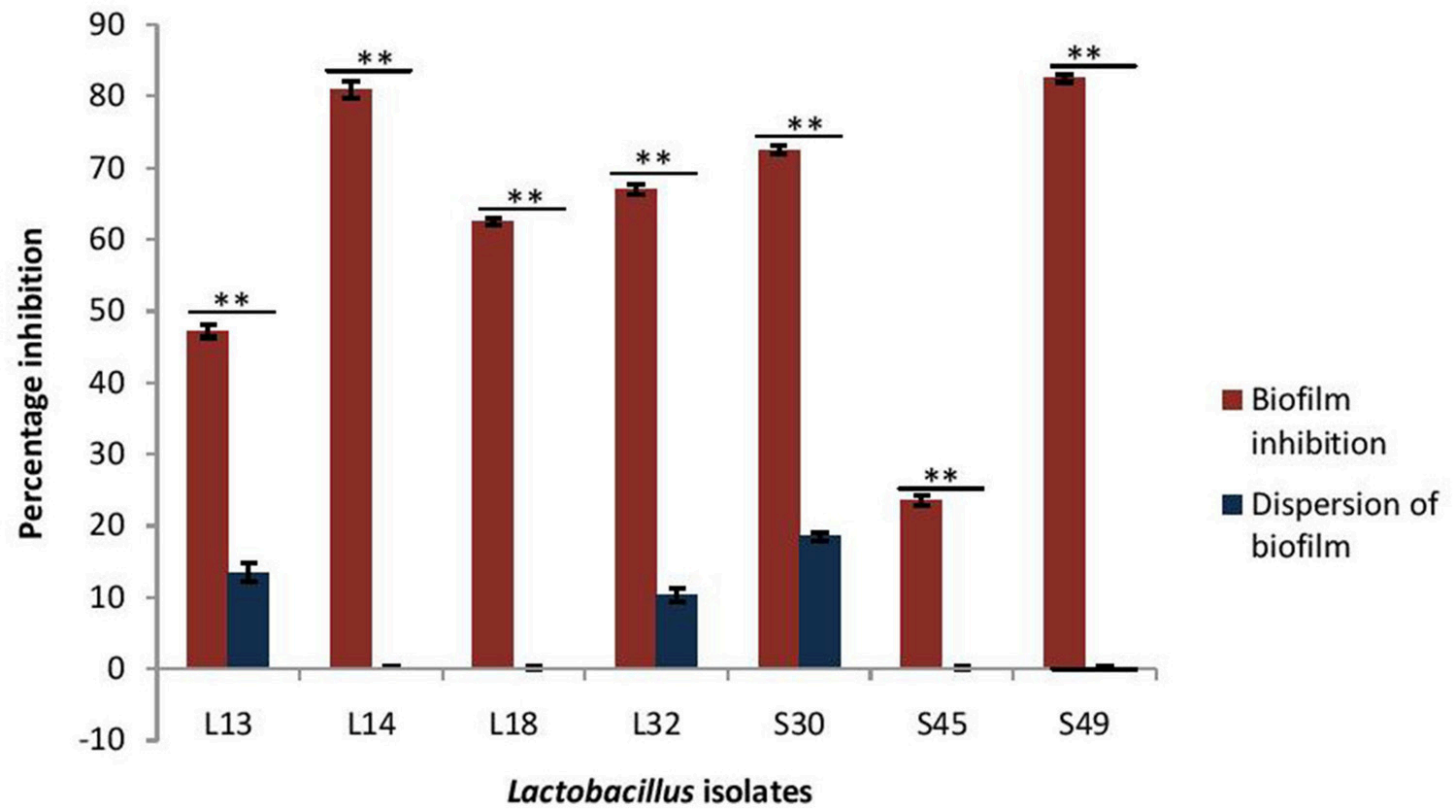
Effect of CS of lactobacilli isolates on the biofilm formation and dispersion of 24 h old performed biofilms of *V. parahaemolyticus*. Bars are representative of the means and error bars represent standard deviation of three independent experiments. ^**^Significance (*p* < 0.0001) was measured by using Student's *t*-test.

As the CS of lactobacilli had no antimicrobial activity against *V. parahaemolyticus*, therefore the inhibitory effects of only pH non-neutralized CS of lactobacilli on the biofilm formation by *V. parahaemolyticus* and its dispersion was determined (Figure 5). The CS of all the isolates, except S45, inhibited the biofilm formation by *V. parahaemolyticus* in the range 47–82%. Maximum inhibition of 82% was observed with the CS of the isolate S49 and L14, followed by S30 (72%), L32 (67%), L18 (62%), and L13 (47%). Interestingly, the CS of all the lactobacilli isolates had no or negligible biofilm-dispersing abilities.

### Effect of CS of lactobacilli on adhesion of *Vibrio* to HCT-15 cell line

**Figure 6:**
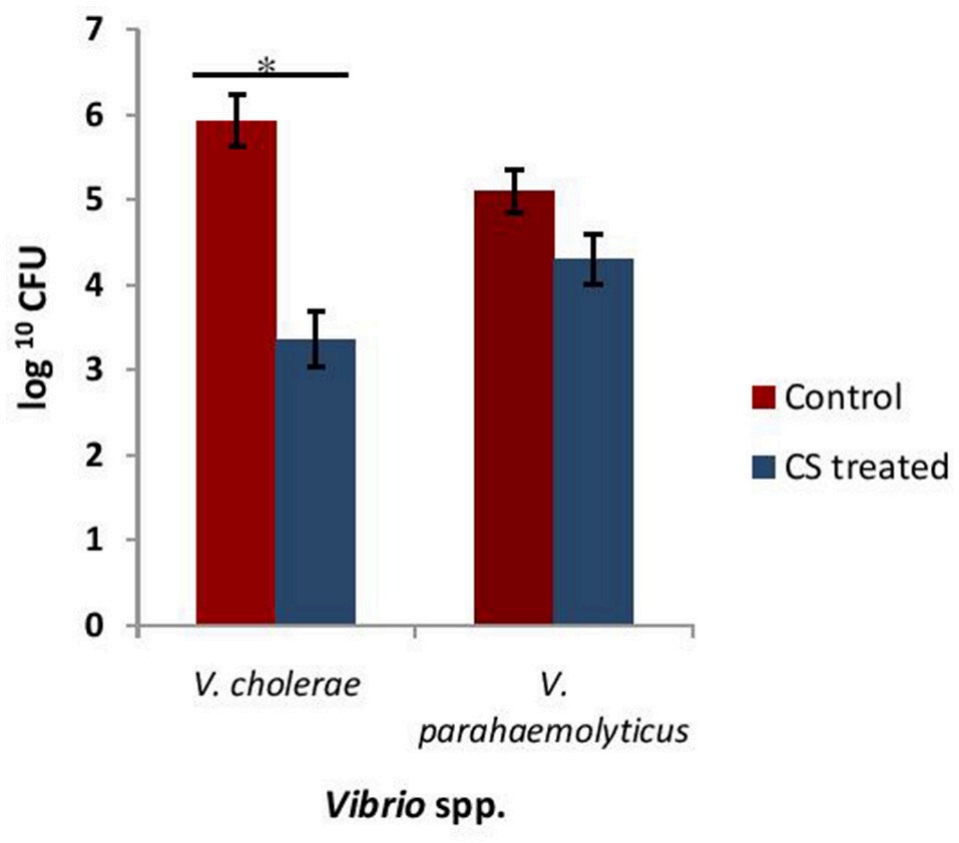
The numbers of CFU of *Vibrio* spp. that adhered to HCT-15 cell line in the absence and presence of CS of L18. Bars are representative of the mean, whereas, error bars are representative of standard deviation of three independent experiments. ^*^Significance (*p* < 0.001) was measured by using unpaired Student's *t*-test.

**Figure 7:**
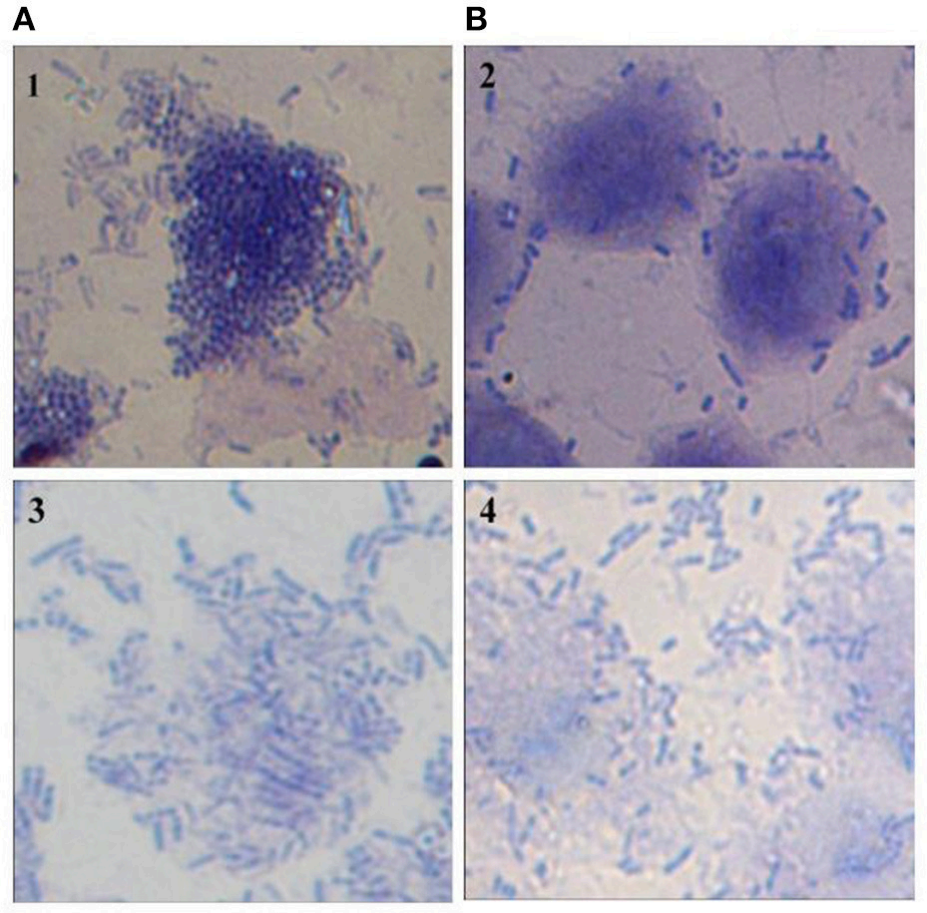
Microscopic (1000X) image of Giemsa-stained HCT-15 cells showing adhered *V. Cholera* (1 and 2) and *V. parahaemolyticus* (3 and 4) in the absence **(A)** and presence **(B)** of CS of lactobacilli isolate L18.

Adherence of *Vibrio* spp. to the intestinal epithelial cells is the primary step involved in the biofilm formation and pathogenesis of gut infection. Therefore, the effect of CS of lactobacilli on the initial adherence of pathogens-*V. cholerae* and *V. parahaemolyticus* with the intestinal cell line HCT-15 was evaluated by both plating technique and by using light microscopy. The binding of *V. cholera* to HCT-15 cell line was almost one log higher as compared to that of *V. parahaemolyticus*. Further, in the presence of CS of L18, there was significant (*p* < 0.001) reduction by approximately 2.5 log_10_ CFUs that adhered to HCT-15. On the other hand, the adherence of *V. parahaemolyticus* in the presence of the CS of L18 was not significantly reduced (Figure 6). The light microscopic images also showed similar effects (Figure 7). The CS of other lactobacilli isolates similarly reduced the adherence of *V. cholera* in the range 1.5–2.2 log_10_CFUs; whereas the adherence of *V. parahaemolyticus* was reduced in the range 0.6–0.9 log_10_CFUs (data not shown).

### Lactic acid production estimation

The lactic acid is the major organic acid produced by glucose fermentation by *Lactobacillus* spp. As biofilm-dispersive abilities in case of L13, L14, L32, and S30 is affected by pH neutralization, we evaluated the concentration of lactic acid in the CS. L13 produced highest amount of lactic acid followed by L32, S49, and L18 whereas S45 and L14 produced least amount of lactic acid (Table 3).

**Table 3:** Percentage of lactic acid produced by lactobacilli isolates.

***Lactobacilli* Isolates**	**% lactic acid produced**
L13	1.60 ± 0.03
L14	1.18 ± 0.02
L18	1.31 ± 0.04
L32	1.42 ± 0.05
S30	1.27 ± 0.02
S45	1.17 ± 0.03
S49	1.40 ± 0.02

*The lactic acid content of the CS was measured after 24 h of growth in MRS broth by titrating against NaOH. The experiment was performed three times in triplicates. The results are expressed as the means ± standard deviations*.

### Probiotic properties of lactobacilli

The probiotic properties of lactobacilli is known to be strain specific, therefore we evaluated the probiotic potential of all the seven lactobacilli isolates. One of the most important criteria for the strain to be good probiotic is its ability to resist the action of bile salts and gastric juice during its passage in the GIT. As expected for the lactobacilli of fecal origin, all the isolates were able to survive after 3 h of incubation in the simulated gastric juice and also showed growth in the presence of bile salts (Table 4).

**Table 4:** Gastric juice and bile juice tolerance of lactobacilli isolates.

***Lactobacilli* Isolates**	**Gastric juice tolerance**	**Bile juice tolerance**
L13	^+++^	^+++^
L14	^++++^	^++++^
L18	^++++^	^++++^
L32	^+++^	^+++^
S30	^++++^	^+++^
S45	^++++^	^++++^
S49	^++++^	^+++^

++++ *, no. of CFUs equal to that obtained in the control well*.

+++ *, 0.2–0.6 log_10_ reduction in the CFUs as compared to control*.

*++, 0.6–2 log_10_ reduction in CFUs as compared to control*.

*+, more than 2 log_10_ reduction in CFUs*.

–*, no growth*.

The ability of lactobacilli to form strong biofilms has been correlated with their abilities to persist *in vivo*. Therefore the abilities of the lactobacilli to form biofilms was evaluated at two different pH 4 and 6, and at different time intervals −24, 48, and 72 h. At pH 4 all the isolates except S45 and S49, formed strong biofilms at 48 and 72 h. Whereas, at pH 6, all the lactobacilli isolates formed strong biofilms after 48 h, but by 72 h biofilms in all except L13 and L18 appeared to disperse and become moderate. L13 and L18 formed strong biofilms at all pH and time points (Table 5).

**Table 5:** Biofilm formation by lactobacilli isolates at different pH and time points using crystal violet microtiter plate assay.

**Lactobacilli Isolates**	**pH 4**			**pH 6**		
	**24 h**	**48 h**	**72 h**	**24 h**	**48 h**	**72 h**
L13	S	S	S	S	S	S
L14	M	S	S	S	S	M
L18	S	S	S	S	S	S
L32	M	S	S	M	S	M
S30	S	S	S	M	S	M
S45	W	W	W	W	S	M
S49	W	W	W	S	S	M

*S, Strong biofilm producers; M, Moderate biofilm producers; W, Weak biofilm producers*.

*The lactobacilli biofilms were formed in MRS media of different pH in 96-well microtitre plates and analyzed at different time points by using crystal violet assay. Based on the OD_595_ of the wells, strains were classified as non-biofilm producers (OD ≤ OD_C_); weak (OD_C_ < OD ≤ 2 × OD_C_); moderate (2 × OD_C_ < OD ≤ 4 × OD_C_) or strong biofilm producers (4 × OD_C_ < OD); where OD_C_ is absorbance of control well having MRS broth with no cells and OD is absorbance of experimental well having MRS broth with lactobacilli cells. The experiment was performed three times in triplicates*.

**Figure 8:**
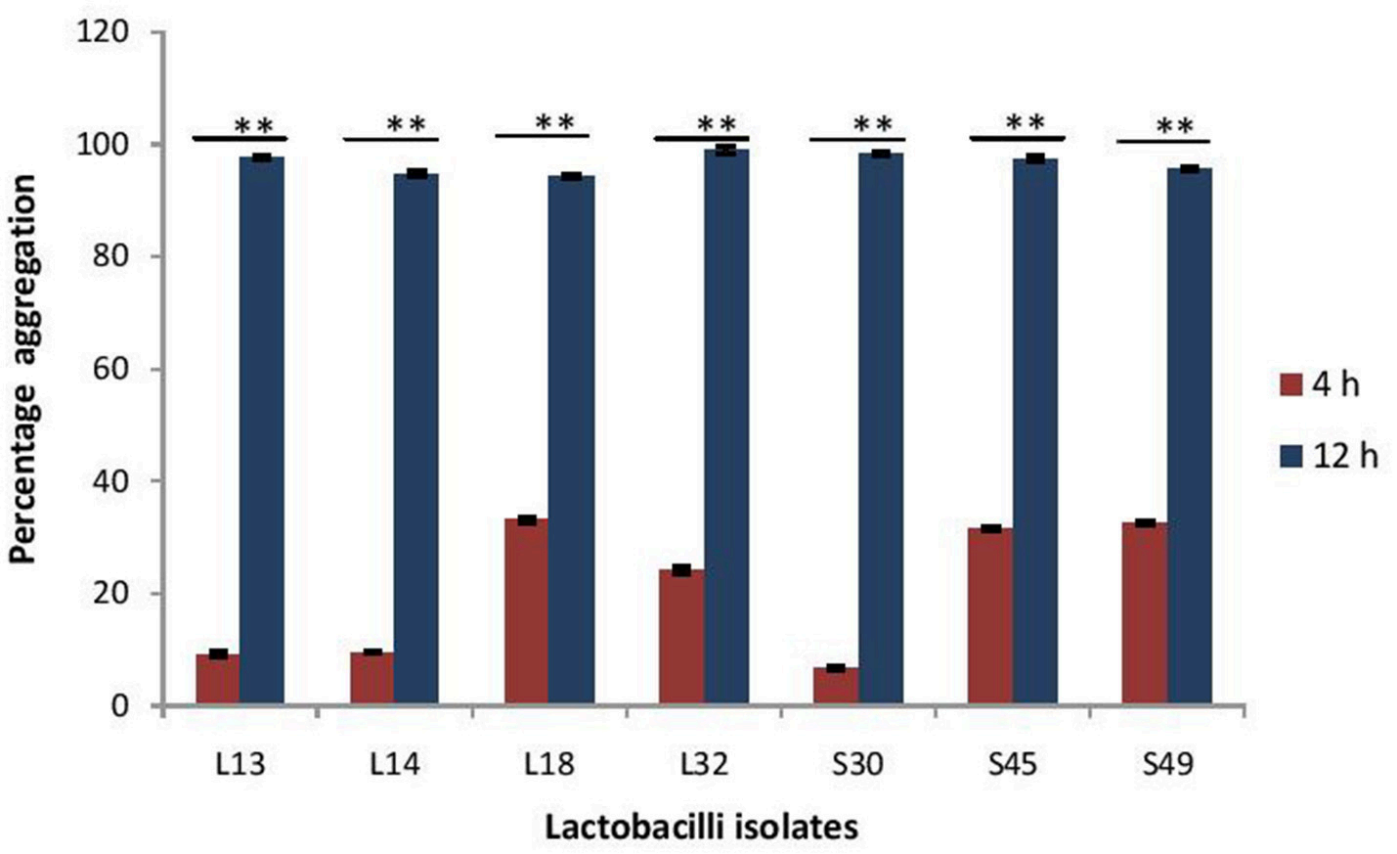
Percentage auto-aggregation exhibited by lactobacilli isolates. Bars represent the mean, error bars represent standard deviation of three independent experiments and ^**^significance (*p* < 0.0001) was measured by using paired Student's *t*-test.

The maturation of biofilms depends on the autoaggregation properties of the lactobacilli. Thus, we assessed their abilities to autoaggregate. All the isolates showed significantly (*p* < 0.0001) higher auto aggregation (more than 90%) after 8 h of incubation that at 4 h (Figure 8).

**Figure 9:**
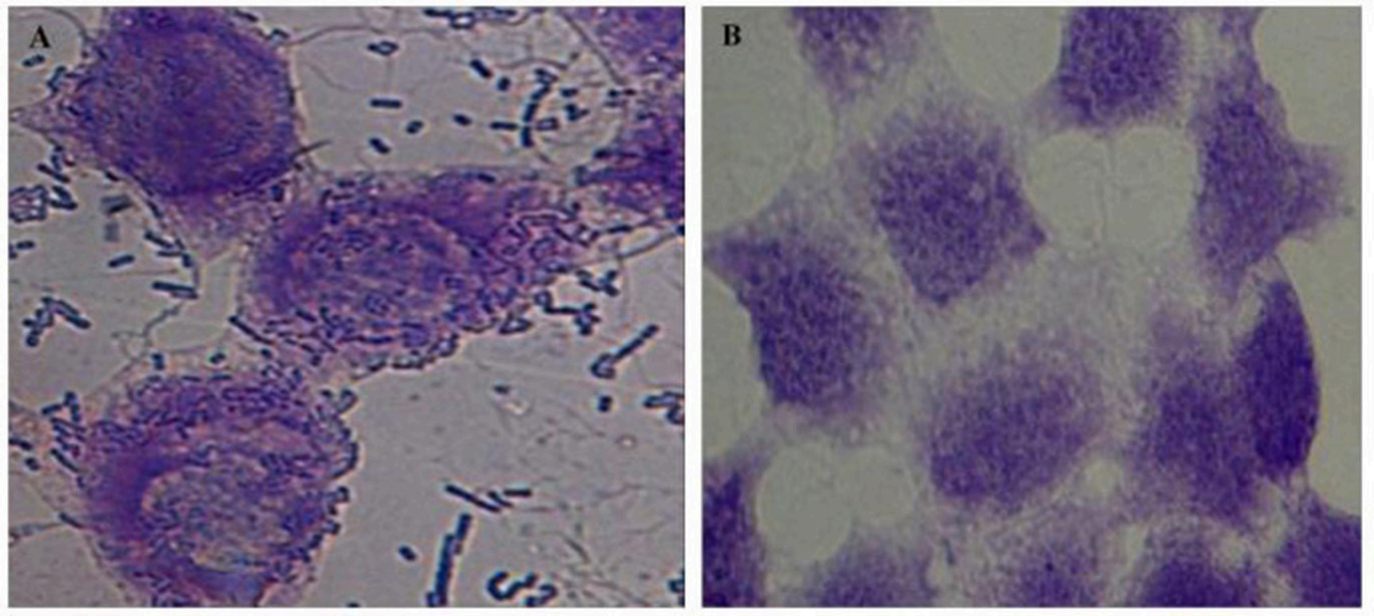
Microscopic (1000X) image of Giemsa-stained intestinal cell line HCT-15 cells with **(A)** and without **(B)** adhered lactobacilli isolate L32.

Further, the ability of the lactobacilli isolates to adhere to the intestinal epithelial cell line HCT-15 was determined. Cell adhesion assay demonstrated that all the selected lactobacilli isolates had strong adhesive ability to intestinal cell line – HCT-15 (Figure 9).

### Antibiotic susceptibility test

Kirby Bauer disc diffusion method was used to analyze the susceptibility profiles of the lactobacilli isolates. Eleven antibiotics belonging to different classes: aminoglycosides (streptomycin, gentamycin, kanamycin), fluoroquinolones (ciprofloxacin, moxifloxacin), beta-lactams (ampicillin, penicillin), macrolides (erythromycin), glycopeptides (vancomycin), lincomycin (clindamycin), and tetracycline (tetracycline), were used in this study. The antibiotic profiles of all the isolates are summarized in Table 6. All of them were susceptible to tetracycline. All, except S45, were susceptible to penicillin and all, except S49, were susceptible to ampicillin, moxifloxacin, and erythromycin.

**Table 6:** Antibiotic susceptibility profile of fecal lactobacilli isolates.

**Antibiotics (conc. in μg)**	**Lactobacilli Isolates**						
	**L13**	**L14**	**L18**	**L32**	**S30**	**S45**	**S49**
TE (30)	S	S	S	S	S	S	S
S (10)	S	I	R	R	S	R	R
CIP(1)	R	R	R	R	R	S	S
MO (5)	S	S	S	S	S	S	I
GEN (10)	S	R	S	S	R	I	R
AMP (2)	S	S	S	S	S	S	R
P (2)	S	S	S	S	S	R	S
VA (30)	R	R	R	R	R	S	S
CD (2)	I	R	I	R	R	R	R
K (30)	R	R	R	R	R	S	R
E (15)	S	S	S	S	S	S	R

*R: Resistant; I: Intermediate; S: Susceptible; TE: Tetracycline; S: Streptomycin; CIP: Ciprofloxacin; MO: Moxifloxacin; GEN: Gentamycin; AMP: Ampicillin; P: Penicillin; VA: Vancomycin; CD: Clindamycin; K: Kanamycin; E: Erythromycin*.

*The antibiotic susceptibilities of lactobacilli isolates were determined by Kirby Bauer method on MRS agar media and the classification as S, I and R was based on the recommended breakpoints for diameters of zone of inhibition as specified by EFSA (2012). The experiment was performed three times in triplicates*.

All the isolates were resistant to clindamycin. All except S45 were resistant to kanamycin and all except S45 and S49 were resistant to vancomycin, and ciprofloxacin. Similarly all, except L13 and S30 were resistant to streptomycin. In case of gentamycin, expect L13, L18, and L32, all were resistant.

### Effect of commercially available drugs on growth of lactobacilli

The effect of commercially available drugs on the growth of lactobacilli was evaluated. Paracetamol, nimugesic, cetrizine HCl, and lansoprazole had no antimicrobial activities against all the lactobacilli isolates. Ibuprofen inhibited the growth of L13, L14, and L18 whereas, diclofenac inhibited the growth of only L14 (Table 7).

**Table 7:** Effect of commercially available drugs on growth of lactobacilli isolates.

**Commercial drug (mg/ml)**	**Zone of inhibition (mm)**						
	**L13**	**L14**	**L18**	**L32**	**S30**	**S45**	**S49**
Paracetamol (500)	–	–	–	–	–	–	–
Diclofenac (10)	–	15 ± 0.2	–	–	–	–	–
Nimugesic (20)	–	–	–	–	–	–	–
Ibuprofen (120)	15 ± 0.1	20 ± 0.2	12 ± 0.1	–	–	–	–
Cetrizine HCl (2)	–	–	–	–	–	–	–
Lansoprazole (4)	–	–	–	–	–	–	–

*The antimicrobial effects of the commercial drugs were determined by agar well diffusion assay. The experiments were performed three times in triplicate. The results are expressed as means ± standard deviations*.

## Discussion

Inhibition of biofilm formation by pathogens is an attractive target for therapeutic intervention (Bjarnsholt et al., 2013) that has received significant attention in recent years, leading to the discovery of biofilm inhibitors for many of the commonly encountered bacterial pathogens including *V. cholera* (Melander and Melander, 2015; Rabin et al., 2015). *V. cholerae* is well known for its ability to form strong biofilms *in vivo* on the intestinal mucosa in rabbit model (Jones and Freter, 1976; Nelson et al., 1976) and in humans (Yamamoto and Yokota, 1988). The experiments conducted in our laboratory also demonstrated strong *in vitro* biofilm-forming abilities of both *V*. *cholerae* and *V. parahaemolyticus* both in 96-well microtite plates and their abilities to bind to HCT-15 colonic epithelial cell line. The ability of *V. cholerae* to develop biofilms is critical to intestinal colonization (Silva and Benitez, 2016) and virulence (Xu et al., 2003; Fong et al., 2010). This is because, biofilm-derived cells could be more effective in competing for limiting nutrients in the small intestine, as suggested by elevated expression of the phosphate uptake system compared to planktonic cells (Mudrak and Tamayo, 2012). Also, biofilms of *V. cholerae* are reported to resist the action of acid inactivation in the gut (Zhu and Mekalanos, 2003). The biofilms once formed become resistant to the action of antibiotics as most of the conventional antibiotics are active only against planktonic *V. cholera* cells and have no biofilm-dispersive action (Warner et al., 2015). Thus, probiotics strains having both antimicrobial and anti-biofilm activities against *V. cholerae* are expected to be clinically superior.

We screened 55 fecal lactobacilli isolates for their antimicrobial potential against Gram-negative gut pathogens, out of which seven isolates having broad-spectrum antimicrobial activities against *V. cholerae, E. coli* and *S. enterica* were selected. Interestingly, the CS appears to inhibit specifically the gut-associated pathogens only and had no antimicrobial action against the commensal gut lactobacilli. However, when pH of the CS was neutralized, the antimicrobial activity was completely abrogated as shown by disappearance of zones of inhibition. This showed that low pH due to lactic acid or other organic acids secreted by the lactobacilli in the CS are responsible for the antimicrobial activities. Similar reports showing abrogation of antimicrobial activities of CS on pH neutralization has been reported earlier also (De Keersmaecker et al., 2006; Zhang et al., 2011). Lactic acid solution at concentration of 0.5% has been shown to effectively kill both Gram-negative (*Salmonella* and *E. coli*) and Gram-positive (*L. monocytogenes*) pathogens by causing cell membrane damage that resulted in leakage of proteins (Qiao et al., 2008; Wang et al., 2015). Thus, lactic acid in the CS (concentrations ranging from 1.17–1.8%) of all the lactobacilli isolates used in the study may be responsible for the antimicrobial activity. However the role of other organic acids for the antimicrobial activity cannot be negated. Also, the catalase enzyme treatment of CS did not altered the zones of inhibition, thereby showing that hydrogen peroxide is not responsible for the antimicrobial activity of CS. Next, the effect of non-neutralized CS on the growth kinetics of *V. cholerae* showed that it had bacteriostatic effects that significantly inhibited the growth till 12 h, beyond which the growth was similar to the wells with MRS. pH neutralized CS on the other hand had no significant effects on the growth kinetics of *V. cholerae*. Similarly, CS of all the lactobacilli strains had no effects on the growth kinetics of *V. parahaemolyticus*.

Further the effects of lactobacilli CS at low pH (3.5) and high pH (after pH neutralization to 6.5) was evaluated on the biofilm-forming ability of *V. cholera* because on pH neutralization the antimicrobial activity of CS is completely abrogated. Results demonstrated that CS at both low and high pH similarly inhibited the biofilm-formation by *V. cholera*. Further, in case of *V. parahaemolyticus* the CS of 5 out of 7 lactobacilli isolates inhibited 62–82% of the biofilm formation despite having no antimicrobial activity. The growth kinetics study also show that both the non-neutralized and the pH neutralized CS did not affect the growth of *Vibrio* at 48 h. Thus, the inhibition of *Vibrio* spp. biofilm formation by lactobacilli CS was not due to its antimicrobial activity. The various components of CS of lactobacilli such as, exopolysaccharides (Kim et al., 2009) and biosurfactants (Walencka et al., 2008; Fracchia et al., 2010; Zakaria Gomaa, 2013) may inhibit the biofilm formation as reported against other pathogens. Purified EPS of *L. acidophilus* was shown to inhibit biofilm formation of a number of Gram-positive and Gram-negative pathogens. It was hypothesized that EPS interfered with the initial attachment of pathogen to small intestine cell line HT-29 (Kim et al., 2009). The physiological pH of mammalian stomach is acidic and that of intestine is toward neutral in the range 6–7. Thus, as the CS of all the lactobacilli isolates inhibited the biofilm formation at both low and high pH, these strains may have good prophylactic action against *Vibrio*-associated infection in both stomach and intestine. However, for the therapeutic action of lactobacilli spp., the dispersive action of CS against the preformed biofilms of *Vibrio* is important.

The CS at low pH dispersed the *V. cholerae* biofilm in the range 62–85%. Even on pH neutralization, the dispersal effect of CS of 6 isolates except L13 was in the range 50–75%; but it was reduced appreciably in the case of 4 isolates. However, none of the CS had any dispersal effects on the biofilm formation by *V. parahaemolyticus*. Thus, apparently the results showed that the biofilm dispersal effect of CS is dependent on its antimicrobial effect. But the role of CS components such as enzymes that caused the disintegration of *V. cholera* biofilm matrix but not that of *V. parahaemolyticus* cannot be negated. The *V. cholera* biofilm matrix is composed of exopolysaccharides containing glucose and galactose as the major components; whereas the biofilm matrix of *V. parahaemolyticus* is made up of capsular polysaccharides that contains many other sugar moieties apart from glucose and galactose (Yildiz and Visick, 2009). Thus, these differences may account for the differential dispersive effect. Further, CS may contain components that induced the secretion of quorum-sensing autoinducer and thereby caused dispersal of *V. cholerae* biofilms preferentially.

Further, we also assessed the abilities of CS of lactobacilli isolates to inhibit the adherence of both *V. cholerae* and *V. parahaemolyticus* to the intestinal epithelial cell line HCT-15. The CS of L18 was more effective at inhibiting the adherence of *V. cholerae* (2.5 log_10_ CFU reduction) to HCT-15 as compared to *V. parahaemolyticus* (0.7 log_10_ CFU reduction). Similar trend was observed with the CS of other isolates. The differences in the viable counts of *V. cholera* and *V. parahaemolyticus* on plating the lysed cell line can be due to the bactericidal action of CS against the *V. cholera* but not against *V. parahaemolyticus*. However, microscopic images of HCT-15 cells showed that L18 CS inhibited the binding of *V. cholera* more than *V. parahaemolyticus*.

Further, the probiotic potential of all the isolates were assessed. For an isolate to be a good oral probiotic candidate, it must have certain survival and adaptive characteristics such as resistance to the low pH, bile salts and various enzymes in the gut. All the *Lactobacillus* isolates showed more than 90% survival in the presence of bile salts and gastric juice. Probiotic bacteria having good biofilm-forming ability can prevent colonization of the gut epithelium by pathogenic bacteria (Jalilsood et al., 2015; Aoudia et al., 2016). Thus, the ability of probiotic bacteria to form biofilms and adhere to colonic cell line was evaluated. All the isolates showed strong adherence to the colonic cell line- HCT-15. Also all the isolates formed strong biofilms at pH-6 after 48 h; whereas, at pH 4 five of the isolates, except S45 and S49 formed strong biofilm. The maturation of biofilms is strongly dependent on the auto-aggregation properties of the probiotic bacteria as it helps the bacteria to form micro-colonies, which in turn secrete exopolysaccharides resulting in the maturation of biofilms. All the lactobacilli isolates used in this study exhibited more than 90% auto-aggregation after 8 h.

As probiotics are commonly used as adjuncts to antibiotic therapy, therefore the inherent resistance of lactobacilli to common antibiotics (Egervärn et al., 2009) was evaluated. Further the antibiotic susceptibility profiles to various classes of antibiotics could provide the hint to the presence of transferable resistance elements that can potentially be transmitted to the gut microbiota (Morelli et al., 1988). All the isolates were susceptible to tetracycline, and all except one were susceptible to erythromycin, ampicillin, penicillin, and moxifloxacin. High ciprofloxacin, gentamycin, streptomycin, and vancomycin resistance were observed among the isolates. The *Lactobacillus* species are known to be intrinsically resistant to vancomycin which is chromosomally-encoded and non-transmissible (Ruoff et al., 1988). Ciprofloxacin resistance in lactobacilli has been reported earlier among lactobacilli of fermented food (Kaktcham et al., 2012) and fecal origin (Shazali et al., 2014). Similar high resistance to aminoglycosides has been reported in probiotic lactobacilli (Zhou et al., 2005) and from fermented food (Kaktcham et al., 2012).

The probiotics are often prescribed along with various drugs such as, analgesic, anti-pyretic, non-steriodal anti-inflammatory drugs (NSAIDs), anti-allergic, and proton pump inhibitor. Therefore, the effect of these commonly used drugs on the growth of lactobacilli isolates need to be evaluated. The drugs - paracetamol, nimugesic, cetrizine hydrochloride and lanzoprazole had no inhibitory effects on the growth of isolates except ibuprofen which inhibited the growth of lactobacilli isolates–L13, L14, and L18 and diclofenac inhibited L14. Previous study also demonstrated the inhibitory effects of sodium diclofenac on the growth of *L. plantarum* ST8KF and ST341LD (Todorov and Dicks, 2008).

All the seven *Lactobacilli* isolates used in this study had broad-spectrum antimicrobial and biofilm-inhibitory activities. Thus, they may have good prophylactic properties to inhibit the gut-associated infectious diseases. The isolates S45, S49, and L18 seemed to possess the best biofilm dispersion abilities at both low and high pH, therefore their therapeutic potential to treat *V. cholera* infection in the mouse model should be further tested.

## Author contributions

SukK conceived the idea and supervised the experiments. SumK and SukK designed the experiments and SumK performed almost all of the experiments, except those involving cell lines. PS, NK, and JS designed and performed the cell line studies. All authors discussed the results and contributed to the final manuscript.

### Conflict of interest statement

The authors declare that the research was conducted in the absence of any commercial or financial relationships that could be construed as a potential conflict of interest.
